# A Safety Computer System Based on Multi-Sensor Data Processing †

**DOI:** 10.3390/s19040818

**Published:** 2019-02-17

**Authors:** Yuan Cao, Hongkang Lu, Tao Wen

**Affiliations:** 1 National Engineering Research Center of Rail Transportation Operation and Control System, Beijing Jiaotong University, Beijing 100044, China; ycao@bjtu.edu.cn; 2 School of Electronic and Information Engineering, Beijing Jiaotong University, Beijing 100044, China; 16120245@bjtu.edu.cn

**Keywords:** safety computer, non-strict multi-sensor identical problems, fuzzy decision tree, fuzzy weighted fusion

## Abstract

The safety computer in the train control system is designed to be the double two-vote-two architecture. If safety-critical multi-input data are inconsistent, this may cause non-strict multi-sensor data problems in the output. These kinds of problems may directly affect the decision making of the safety computer and even pose a serious threat to the safe operation of the train. In this paper, non-strict multi-sensor data problems that exist in traditional safety computers are analyzed. The input data are classified based on data features and safety computer features. Then, the input data that cause non-strict multi-sensor data problems are modeled. Fuzzy theory is used in the safety computer to process multi-sensor data and to avoid the non-strict multi-sensor problems. The fuzzy processing model is added into the onboard double two-vote-two architecture safety computer platform. The fuzzy processing model can be divided into two parts: improved fuzzy decision tree and improved fuzzy weighted fusion. Finally, the model is verified based on two kinds of data. Verification results indicate that the fuzzy processing model can effectively reduce the non-strict identical problems and improve the system efficiency on the premise of ensuring the data reliability.

## 1. Introduction

With the development of the railway field, the safety computer is playing an increasingly important role in the train control system [1,2,3,4]. Due to different data having different data characteristics, the analysis and discussion of multi-sensor data processing in the safety computer are of great significance. The current problem is that analog data of multi-channel sensors are inconsistent due to the sensors’ own features and environmental factors, which leads to non-strict multi-sensor data problems in the safety computer. Because of the increasing amount of data, these problems are becoming more and more serious. They will bring problems such as inefficiency and low availability in train control systems, even affecting the safe operation of the train if these data are safety-critical data, such as speed data. For example, there are multiple inputs of speed data in train control systems. The double two-vote-two onboard safety computer requires two inputs of speed data. If these data are different, the onboard safety computer will switch the system. However, this does not solve the problem because the safety computer still does not have an accurate value of speed data to calculate the operation curve for the next step. Various types of data in the train control system have this kind of problem, such as speed data, air pressure data, and positioning data.

Yu, H.J. proposed a weighted fusion method in speed data [5]. Meng, X.Y. simulated and modeled the air pressure data [6]. There has been some research on improving data accuracy in the railway field. However, few research works connect analog data and the non-strict multi-sensor data problems in the safety computer. Although there is no effective solution nor breakthrough in the railway field for multi-sensor data problems, the research on multi-sensor data problems has made many important achievements in other fields. In the field of nuclear power, Huang, Q. proposed a fuzzy idea to process multiple input data and achieved certain results [7]. Zhang, S.C. introduced fuzzy theory to data processing to reduce the influence of multiple environmental factors [8]. In the aerospace field, Liu, Q. improved the multiple input algorithm to meet the development of the aviation industry [9]. Dong, Y. processed analog data separately according to the different acquisition methods for the different analog data [10]. However, both fields researched the architecture of three-out of-two and four-out of-three and only focused on improving data accuracy without considering the architecture characteristics. None of them has ever researched the architecture of double two-vote-two. This paper focuses on both architecture characteristics and multiple analog data. Fuzzy theory is used in the safety computer to improve data accuracy. Unlike traditional methods, it has the advantages of solving the non-strict multi-sensor data problems and improving the safe operation of the train control system.

In this paper, an improved fuzzy processing model based on the fuzzy theory and the onboard safety computer is proposed. It can be divided into two parts: improved fuzzy decision tree and improved fuzzy weighted fusion. The traditional fuzzy decision tree based on the fuzzy iterative dichotomizer 3 (FID3) algorithm has a poor real-time data processing ability and cannot analyze the historical data. Therefore, an improved real-time FID3 algorithm based on historical data is proposed, which can effectively process real-time data and preliminarily filter data according to historical data and the membership function. An improved fuzzy weighted fusion model introduces fuzzy mathematics into the traditional weighted fusion model, with the advantages of high fusion precision and strong robustness. Fuzzy weighted fusion can solve nonlinear problems and environmental disturbance. Introducing fuzzy theory into the safety computer can not only solve the non-strict multi-sensor data problem, but also improve the accuracy and robustness of data. This paper proposes an improved safety computer model that processes data separately to improve the data processing capability and system efficiency.

The rest of this paper is organized as follows. Section 2 introduces the non-strict multi-sensor data problems in the safety computer and classifies the data according to the data features. This paper proposes an improved onboard computer platform. Section 3 introduces data feature extraction and two kinds of fuzzy algorithms used in the fuzzy processing model. Section 4 analyzes and simulates the established model, and the conclusions are given in Section 5.

## 2. The Introduction of the Safety Computer into the Train Control System

### 2.1. Non-Strict Multi-Sensor Data Problems in the Safety Computer

The traditional double two-vote-two safety computer compares the homogenous and heterogeneous redundant data during data processing, and the redundant data need to be identical. If these data are different, the safety computer will switch regardless of the slightness of the data difference. Although this logical frame can ensure system safety, it may cause the problem of data inconsistency if input data are analog data, such as speed data and air pressure data, which require accurate values. The problem cannot be solved by switching the system. Safety-critical analog data play a vital role in the operation of the train control system. If these data have errors, this will bring problems such as inefficiency and low availability, even affecting the safe operation of the train.

**Figure 1:**
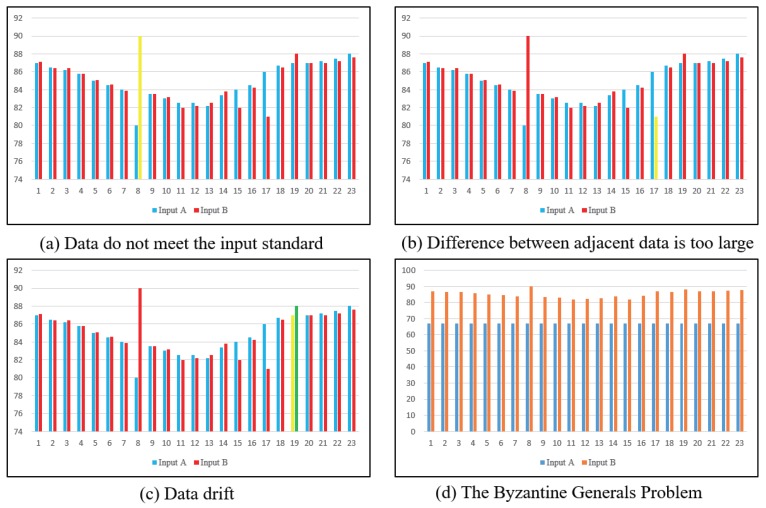
The architecture of the improved onboard safety computer platform.

The problems of multi-sensor data inconsistency can be roughly divided into four cases, i.e., the data parameters do not meet the input standard, there is a large difference between adjacent data, data drift, and the Byzantium problem. (1) The data parameters that do not meet the input standard mean that the values of the collected data far exceed their standard range. For example, the maximum train speed can reach 350 km/h, but the collected value is 600 km/h. In this case, the data input should be discarded. (2) The difference between adjacent acquisition data is obviously too large, which is obviously a sensor failure. If the data are input to a safety computer for data processing, there will be a situation where the efficiency is reduced and resources are wasted. (3) After the multi-channel sensor collects data, data drift may occur due to channel interference and environmental interference. (4) The Byzantium problem is a common problem in fault tolerance mechanism. It is impossible to achieve consistency by means of messaging on an unreliable channel with data failure. Figure 1 shows four kinds of problems of multi-sensor data inconsistency.

Currently, in the train control system, the method of calculating the average value for multiple input data is adopted. However, air pressure data are affected by many factors like pipe diameter, duct shape, and the hysteresis effect. In this case, the method is obviously unsafe for the operation of the train control system.

### 2.2. Data Features’ Discussion

As a safety critical system, the safety computer has different requirements for different data. According to the principle of safety, periodicity, redundancy, and functionality, the data can be divided into three types: state data, dynamic data, and static data.

State data are safety-critical data, and this reflects a certain state of the device or track in the operation of the train control system, such as the state of the segment. This type of data is usually digital data.

Dynamic data such as speed data and air pressure data are analog data which are required for calculation in the operation of the train control system. They play a vital role in system operation, which has a strong real-time performance and requires accurate values.

Static data are mainly composed of monitoring information and log information that have a large amount of data and low safety.

The non-strict multi-sensor data problems of the safety computer can only be caused by dynamic data. Therefore, modeling dynamic data separately can effectively improve the data processing capability and system efficiency. Establishing a dynamic database requires a large amount of dynamic data. A feature parameter database can be obtained by extracting features from the data. It is worth noting that data features need to be classified and extracted according to different operating environments and different operating modes.

### 2.3. The Architecture of the Safety Computer in the Train Control System

Traditional onboard safety computer platforms adopt the architecture of double two-vote-two. This kind of architecture forms a double relationship between two computers with identical functional structures, and each computer has two heterogeneous units to form the relationship of two-vote-two [11].

Only the dynamic data can result in the non-strict multi-sensor data problems of the safety computer. Thus, a fuzzy processing model based on dynamic data is established. Under the architecture of double two-vote-two, this paper proposes an improved onboard safety computer platform, which adds a fuzzy processing model to process dynamic data separately to improve the system communication efficiency and capability of data processing.

**Figure 2:**
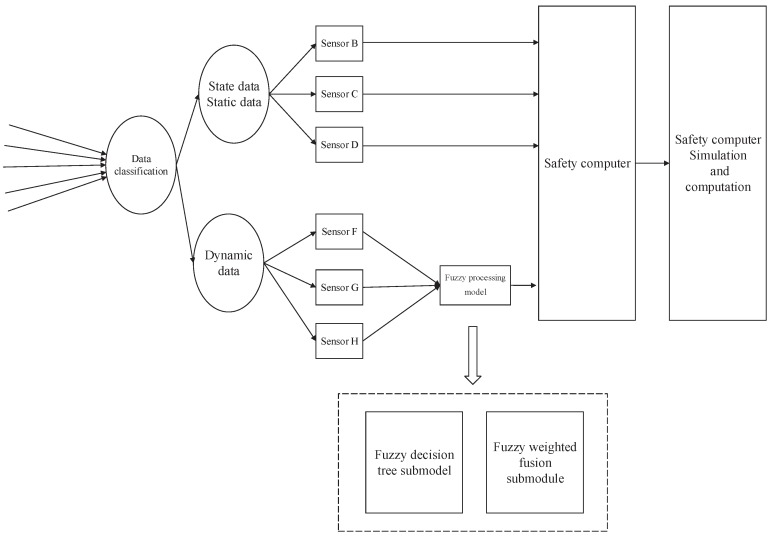
The architecture of the improved onboard safety computer platform.

The fuzzy processing model consists of two parts: improved fuzzy decision tree and fuzzy weighted fusion. The architecture of the improved onboard safety computer is shown as Figure 2.

## 3. The Introduction of Fuzzy Theory

### 3.1. Data Feature Extraction and Selection

The extraction and selection of data features play important roles in data classification, algorithm calculation, and simulation verification [12]. The data of train control system are characterized by a large amount of data, high repetition, and contradiction. It is very difficult to extract the information needed from the massive amount of data, so the data features of the train control system must be preprocessed in order to accurately reflect the state of the system.

The steps of data feature extraction are sorting, grouping, collating, dimensionality reduction, and illustration. The function of these steps is to further understand the distribution features of data and then find out the representative values that reflect the distribution features of data.

The feature distribution of data is described in three aspects: the centralized trend of data distribution, the discrete degree of data distribution, and the shape of data distribution. The centralized trend of data distribution reflects the degree of convergence or the convergence of data toward their central value; the discrete degree of data distribution reflects the trend of data away from their central value; and the shape of data distribution reflects the skewness and peak state of the data distribution.

Data feature extraction is the basis of the algorithm, which determines the result of algorithm simulation and the authenticity of the experiment. Therefore, data extraction needs to refer to a variety of factors affecting data, such as different operating modes, operating environments, and sensor types.

Different data features are extracted for different purposes. For example, the average value reflects the centralized trend of the data, and the variance reflects the degree of discreteness of the data.

**Figure 3:**
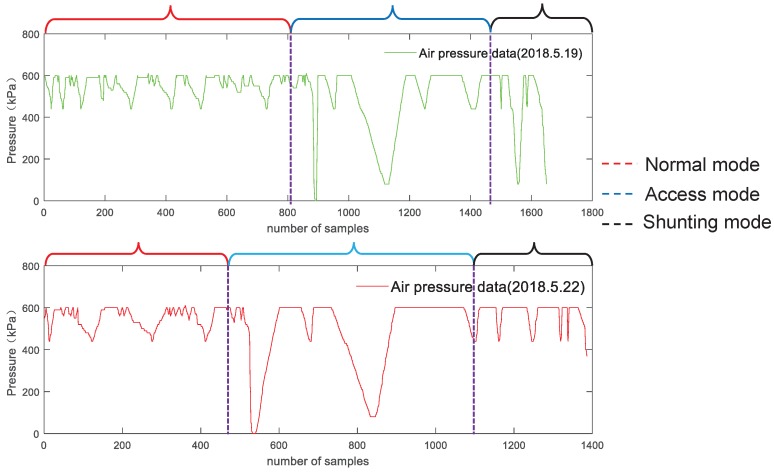
The architecture of the improved onboard safety computer platform.

It is incomplete to use a single data feature to reflect the whole system state, so this paper extracts several data features in data processing, and two kinds of data features are selected from each of three aspects of data features. The centralized trend of the data distribution chooses the mean value and median; the discrete degree of the data distribution chooses the standard deviation and discrete coefficient; and the shape of the data distribution chooses skewness and peak state. It is worth noting that the same kind of data collected from different sensors may also be different data feature. In data weighted fusion, the weights of different sensors need to be considered in particular. Figure 3 shows three operation modes of air pressure data.

### 3.2. The Improved Fuzzy Decision Tree

The fuzzy decision tree introduces fuzzy mathematics into a classical decision tree. The difference between the classical decision tree and fuzzy decision tree is that the fuzzy decision tree needs to establish the membership function to represent the membership degree of data. The fuzzy decision tree use fuzzy multi-valued logic with ambiguous values instead of binary logic with precise numerical values.

The establishment process of the fuzzy decision tree model can be divided into six steps: (1) data collection; (2) data preprocessing; (3) induction and establishment of the fuzzy decision tree; (4) pruning the established fuzzy decision tree; (5) converting the fuzzy decision tree into a set of fuzzy rules; (6) applying the obtained fuzzy rules to forecast [13,14,15].

The fuzzy decision tree based on the FID3 algorithm has a poor real-time data processing ability and cannot analyze the historical data. Aiming to deal with these disadvantages, this paper proposes an improved real-time FID3 algorithm based on historical data, which fully takes historical data and real-time performance into consideration. Data preprocessing is one of the most important steps in the modeling of the fuzzy decision tree. The function of data preprocessing is to fuzzify the collected data and establish its membership function. In this paper, the membership function is established according to the speed data. In addition, a time function and a delay parameter are added to satisfy the requirements of historical data and real-time performance. The time function can be divided into two parts, the historical credibility and a similarity function. The function of the equation is to compare the similarity of adjacent data. Different data have different membership functions. Speed data will be used to build the improved fuzzy decision tree. The delay parameter represents the time delay of the data.

According to the characteristics of the data, the algorithm adds a time function based on the initial one. Assume there is an input instance $U=\{\left(U_{1},t_{1}\right),\left(U_{2},t_{2}\right),\cdots ,\left(U_{N},t_{N}\right)\}$. For each example $\left(U_{i},t_{i}\right)$, the membership function for the *j* attribute of the variable $U_{i}$ is $\mu_{ij}\left(t_{i}\right)$, as shown in Formula (1).

(1) $$\mu_{ij}\left(x,t_{i},\Delta t\right)=\left\{\begin{array}{cc} \;0\; & x>0,g\left(t_{i}\right)<0.5,\Delta t>t_{max}\\ e^{\frac{x-\delta }{\sigma T(t_{i})}}\; & x>0,g\left(t_{i}\right)>0.5,\Delta t<t_{max} \end{array}\right.$$

(2) $$\delta =V\left(t_{i}\right)-V\left(t_{i-1}\right)-\sum_{j=i-1}^{i}t_{i}a_{j}$$

In the formula, $t_{i}$ denotes the data acquisition time. σ denotes the distribution parameter. $g(t_{i})$ denotes the time function. δ denotes the difference between the speed acquisition value and the speed estimation value. $V(t_{k})$ denotes the speed data collected at $t_{k}$ point. $\sum_{j=i-1}^{i}t_{j}a_{j}$ denotes the speed change value calculated by the collected acceleration. The abscissa xdenotes the historical speed value and the range of values is [0,+∞]. Δt denotes the maximum delay allowed for the system.

(3) $$T\left(t_{i}\right)=\theta \left(t_{i}\right)\beta_{t_{i}}$$

(4) $$\theta \left(t_{i}\right)=1-\frac{(\sum_{k=1}^{M}|\left(U_{i},t_{i}\right)-\left(U_{i-1},t_{i-1}\right){{|}^{2})}^{\frac{1}{2}}}{\Delta_{MAX}}$$

In the formula, $T(t_{i})$ denotes the degree of real-time and the reliability of the data collected at time $t_{i}$, and the value’s range of $T(t_{i})$ is [0,1]. $\theta (t_{i})$ denotes the similarity of data collected at times $t_{i-1}$ and $t_{i}$. The value’s range of $\theta (t_{i})$ is [0,1]; the higher the similarity is, the closer to one. $\beta_{t_{i}}$ denotes the historical credibility of sensor, which ranges from [0,1]. The higher the reliability is, the closer to one.

The rest of the algorithm is similar to the FID3 algorithm. According to the principle of maximum information gain, each time, the attribute with the greatest information gain is selected as the test attribute. In the process of dividing, according to the value of the degree of membership of a certain test attribute of each instance, it can be subsumed into multiple subsets to generate a fuzzy decision tree.

The functions of the improved fuzzy decision tree are data fuzzification, the generation of historical credibility, and data preliminary filtering.

### 3.3. Fuzzy Weighted Fusion Algorithm

Different sensors have different operational principles. Different sensors have their own advantages under different environmental conditions. The weighted fusion model gives different weights to different sensors; however, it is difficult to ensure the accuracy of the fusion for real-time and variable data. Fuzzy weighted fusion introduces fuzzy mathematics into the traditional weighted fusion model, with the advantages of high fusion precision and strong robustness. Fuzzy weighted fusion can solve nonlinear problems and environmental disturbance.

**Figure 4:**
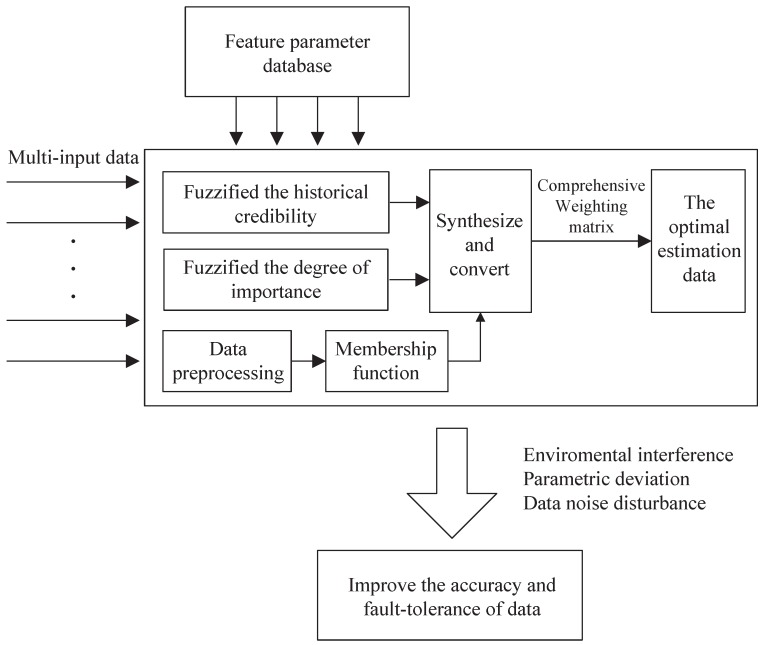
The architecture of the improved onboard safety computer platform.

The main function of the fuzzy weighted fusion algorithm is to fuse multi-sensor data and improve the accuracy of data and fault tolerance. The degree of importance and historical credibility are used in the algorithm for the problem of different sensor sensitivities under different environmental conditions. The main point of this fuzzy weighted fusion algorithm is to fuzzify the degree of importance and then convert the fuzzified degree of importance and historical credibility into a comprehensive weighting matrix. Finally, the multi-sensor data are fused by a comprehensive weighting matrix. Figure 4 shows the structure of the fuzzy weighted fusion algorithm.

Assuming there are multiple inputs $U=U\{U_{1},U_{2},\cdots ,U_{m}\}$, $S_{i}$ denotes the historical credibility, and $P_{i}$ denotes the degree of importance [16,17,18,19].

The fuzzy weighted fusion algorithm is divided into five steps: (1) the fuzzification of the importance degree and historical credibility; (2) the analysis of the importance degree and historical credibility fusion; (3) determining the fuzzy attribute value of the data; (4) the weighted fusion of data; (5) the simulation and verification on the fuzzy weighted data.

The first step is to fuzzify the importance degree and the historical credibility. The historical credibility is fuzzified in the improved fuzzy decision tree. The fuzzification of the importance degree is based on the feature parameter database according to different environments and operating modes.

The second step is to analyze the historical credibility and importance degree after fuzzification. OWA (ordered weighted averaging operator) is used in the transformation. OWA is that between the maximum and minimum operators. It can be used to fuse multi-source and multi-attribute data effectively by adjusting the degree or-and in the operator.

There are two measures associated with an OWA operator, orness and andness.

(5) $$orness\left(w\right)=\frac{1}{n}\sum_{i=1}^{n}\left(n-i\right)w_{i}$$

(6) andness(w)=1−orness(w)

In the formula, *n* denotes the number of data. Wdenotes an ordered weighted vector.

An ordered weighted vector $w=w(w_{1},w_{2},\cdots ,w_{n})$ can be defined as:

(7) $$w_{i}=Q\left(\frac{i}{n}\right)-Q\left(\frac{i-1}{n}\right)$$

In the formula, *Q* denotes a fuzzy semantic quantization operator, which can be defined as:

(8) $$Q\left(r\right)=\left\{\begin{array}{cc} 0\; & r<\alpha \\ \frac{r-\alpha }{\beta -\alpha } & \alpha \leq r\leq \beta \\ 1 & r>\beta \end{array}\right.$$

In the formula, the value range of α,β,r is [0,1].

The parameters α,β are affected by the characteristics of the sensor, environmental factors, and different operating modes.

The third step is to synthesize and convert the historical credibility and importance degree. The fuzzy synthesis algorithm is used in transformation; a fuzzy conversion operator is defined as:

(9) $$\left\{\begin{array}{c} G_{max}\left(P_{i},S_{i}\right)=T\left(P_{i},S_{i}\right)\\ G_{min}\left(P_{i},S_{i}\right)=S\left(\bar{P_{i}},S_{i}\right)\\ G_{ave}=\frac{m}{d}P_{i}S_{i}\\ d=\sum_{i=1}^{m}P_{i} \end{array}\right.$$

In the formula, *m* denotes the number of data sources. *d* denotes the sum of the importance degree. *T* and *S* denote two different fuzzy logic operators.

Based on the previous discussion, fuzzy synthesis function can be defined as:

(10) $$\begin{array}{cc} G= & (high\left(c\right)G_{max}+medium\left(c\right)G_{ave}\\ & +low\left(c\right)G_{min}/high\left(c\right)\\ & +medium(c)+low(c)) \end{array}$$

The rules are as follows:

- If orness(w) is high, then *G* is $G_{max}$;
- If orness(w) is medium, then *G* is $G_{ave}$;
- If orness(w) is low, then *G* is $G_{min}$;

Assuming that $R_{i}=G\left(P_{i},S_{i}\right)$, $R_{i}$ denotes the fuzzy attribute value of the *i*th data after fuzzy synthesis. $G_{max},G_{ave}$, and $G_{min}$ can be defined as:

(11) $$\left\{\begin{array}{c} G_{max}=P_{i}S_{i}\\ G_{ave}=\frac{m}{d}P_{i}S_{i}\\ G_{min}=1-P_{i}+P_{i}S_{i} \end{array}\right.$$

In the fourth step, the sensor state matrix can be calculated by the weighted fusion formula that is defined as follows:

(12) $$C=E^{*}\times W^{\prime }=\sum_{i=1}^{n}E_{i}^{*}\times R_{i}$$

In the formula, *C* denotes the fusion data. *t* denotes multi-source data collected by real-time multi-channel sensors.

The last step is to verify the algorithm. The dynamic data are divided into two parts after collection, the test set and training set. The training set is used for modeling, and the test set is used to verify the model. The model needs to be verified in multiple operating modes because data from different operating modes have different features.

## 4. The Modeling of the Fuzzy Processing Model

### 4.1. Fuzzy Decision Tree Submodel

The fuzzy processing model is capable of processing multiple dynamic data. Two kinds of dynamic data (speed data and air pressure data) will be used in this section. The speed data in this paper were acquired by the wheel speed sensor and GPS sensor of the laboratory test vehicle. The air pressure data were collected in real time from the train K9828 in Yunnan Province.

**Figure 5:**
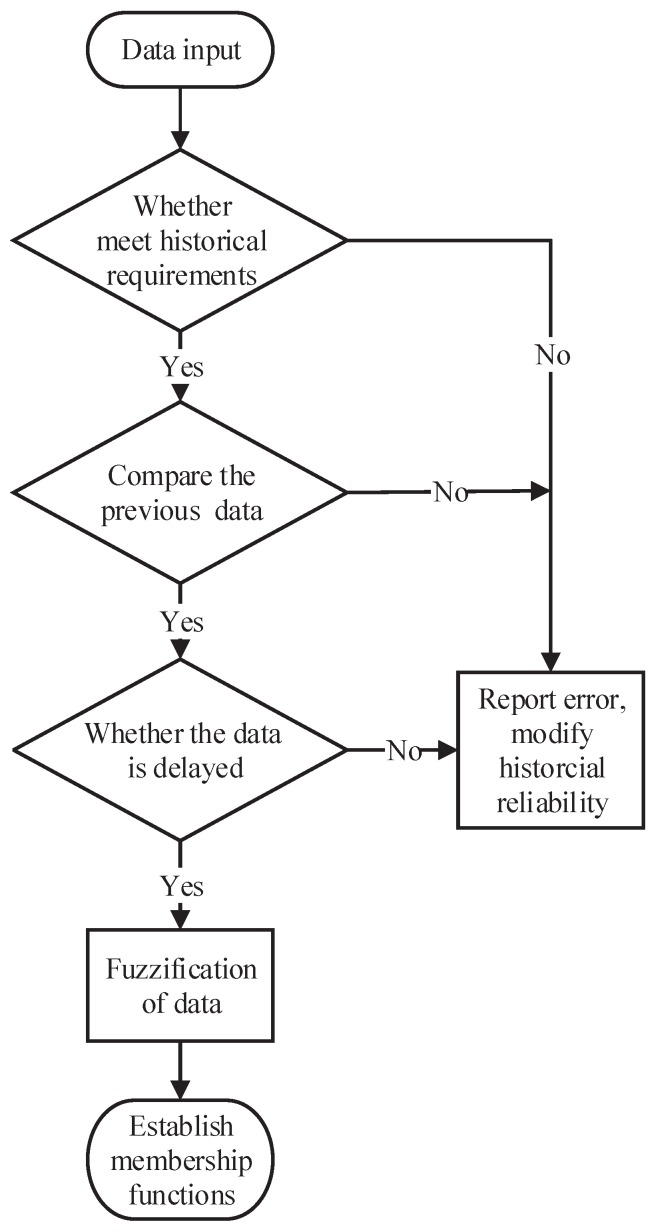
The architecture of the improved onboard safety computer platform.

Before data fuzzification, all the data need to be reprocessed, as shown in Figure 5. This can be divided into four steps.

Step 1: The data are input into the historical database. If the historical range is satisfied, turn to Step 2; otherwise, modify the historical credibility.
Step 2: Calculate the simulation speed based on the previous frame speed data and acceleration. The difference between the simulation speed and current frame speed are obtained. If the difference has a reasonable range calculated according to historical data, turn to Step 3; otherwise, modify the historical credibility
Step 3: If data are delayed, modify the historical credibility; otherwise, turn to Step 4.
Step 4: Fuzzify the data to establish membership functions based on slope parameters, environmental factors, and acceleration information.

**Figure 6:**
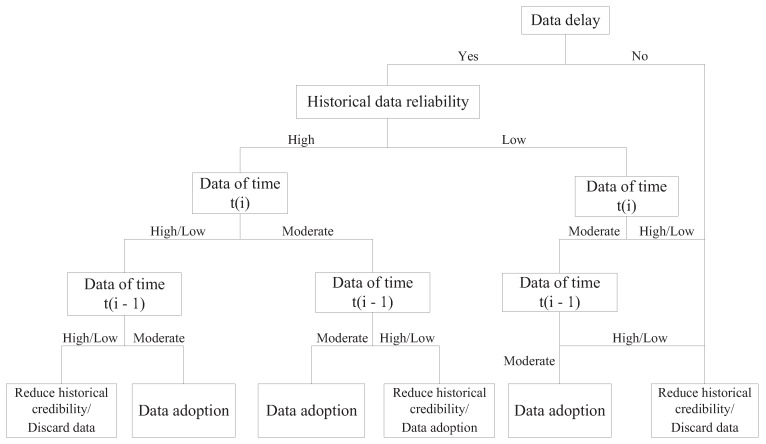
The architecture of the improved onboard safety computer platform.

Using the proposed algorithm, the feature properties of all data gains are examined. Then, the fuzzy set is segmented and iterated. Finally, a fuzzy decision tree is obtained, as shown in Figure 6. This algorithm uses speed data as an example to model.

The fuzzy rules can be extracted by the established fuzzy decision tree. There are five parts to the fuzzy rules (the degree of confidence of the rules):

- IF the data are not delayed AND high historical credibility AND moderate real-time speed AND moderate historical speed, THEN speed adoption (0.97);
- IF data are not delayed AND high historical credibility AND moderate real-time speed AND low/high historical speed, THEN speed adoption (0.93);
- Data are not delayed AND high historical credibility AND low/high real-time speed AND moderate historical speed, THEN speed adoption (0.94);
- IF Data are not delayed AND high historical credibility AND low/high real-time speed AND low/high historical speed, THEN discard speed (0.92);
- IF Data are delayed, THEN discard speed (1);

### 4.2. Fuzzy Weighted Fusion Submodule

The multiple inputs of speed data need to be weighted and merged to improve the data accuracy by the fuzzy decision submodule. This submodule adopts the feature parameter database and the fuzzy weighted fusion algorithm introduced in the previous section. The fuzzy level can be defined as:

(13) $$\left\{\begin{array}{cccc} high(c)=0 & c\leq 0.5\\ high(c)=2c-1 & c>0.5 & medium(c)=2c & c\leq 0.5\\ medium(c)=2-2c & c>0.5 & low(c)=1-2c & c\leq 0.5\\ low(c)=0 & c>0.5 \end{array}\right.$$

If c≤0.5, $R_{i}$ can be defined as:

(14) $$R_{ij}=G_{i}=\left(1-2c\right)\left(1-P_{i}+P_{i}S_{i}\right)+2c\left(\frac{m}{d}P_{ij}S_{i}\right)$$

If c>0.5, $R_{i}$ can be defined as:

(15) $$R_{ij}=G_{i}=\left(2c-1\right)P_{ij}S_{i}+\left(2-2c\right)\left(\frac{m}{d}P_{i}S_{i}\right)$$

### 4.3. Algorithm Analysis and Verification

The functions of the fuzzy decision tree submodule are data fuzzification and data preliminary filtering on multiple inputs of data and adjusting each input’s historical credibility based on whether the data meet the input criteria. Currently, the train control system adopts a redundant structure to collect data, so four channels of speed data are collected (A, B, C, D).

**Figure 7:**
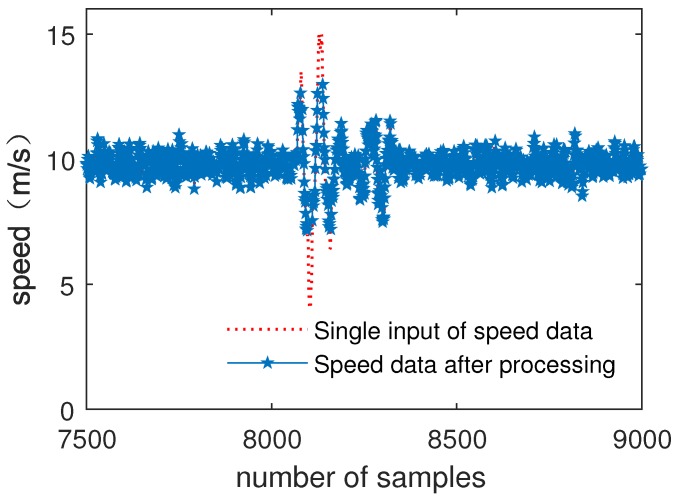
Single input of speed data and processed data.

Figure 7 shows the comparison of Channel A data and data processed by the fuzzy decision tree submodule.

**Figure 8:**
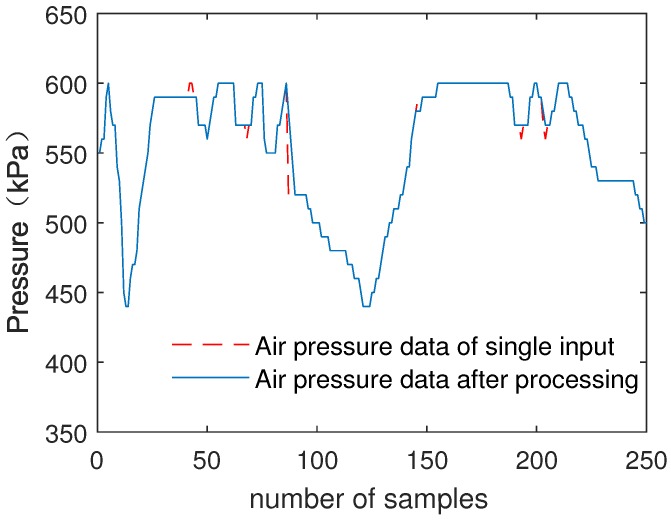
Single input of air pressure data and processed data.

Figure 8 shows the comparison of the single input of air pressure data and data processed by the fuzzy decision tree submodule.

As can be seen from Figure 7 and Figure 8, the noise of air pressure data is preliminarily filtered out. The results show that fuzzy decision tree submodule improves data reliability and data accuracy.

In order to verify the reliability of the model, fault data are randomly injected into the test set of speed data and air pressure data. Then, the test set data are input into the fuzzy decision tree submodule.

**Figure 9:**
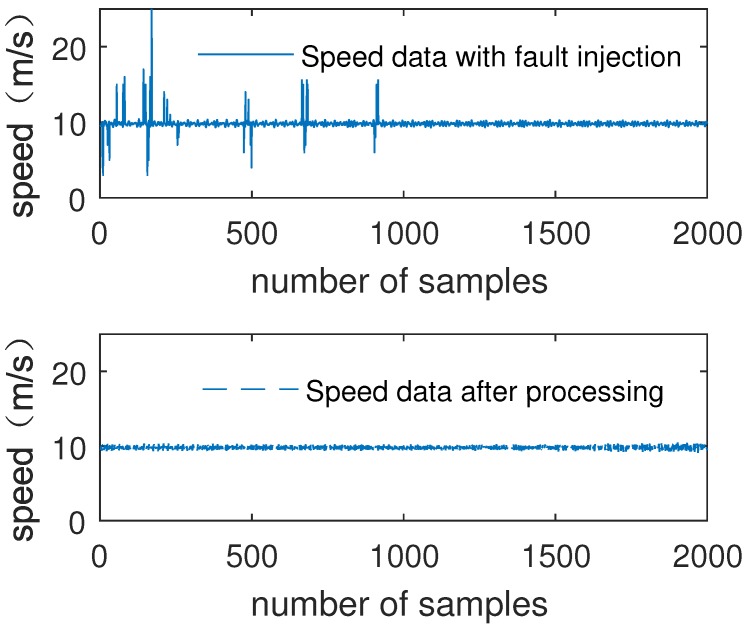
Single input of speed data with fault injection and processed data.

Figure 9 shows the comparison of the single input of speed data with fault injection and data processed by the fuzzy decision tree submodule.

**Figure 10:**
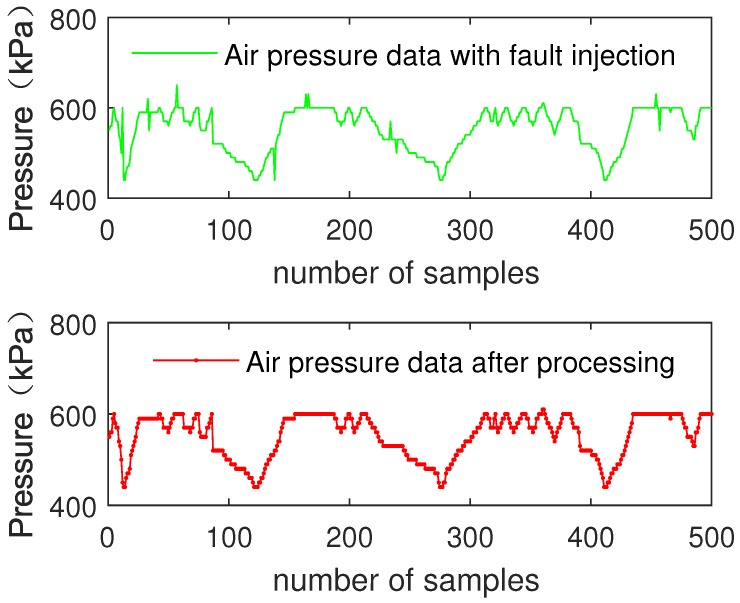
Single input of air pressure data with fault injection and processed data.

Figure 10 shows the comparison of the single input of air pressure data with fault injection and data processed by the fuzzy decision tree submodule.

As can be seen from Figure 9 and Figure 10, after submodule processing, no matter the data’s noise level, the injected fault data of air pressure data and speed data are eliminated. The results show that the fuzzy decision tree submodule effectively improves the data inconsistency and improves the reliability of the data.

The simulation results of MATLAB show that the accuracy of speed data is increased by 15.72%, and air pressure data is improved by 17.57%. For injection faults, the elimination rate is 100%.

There are four channels of speed input data in the train control system because single input data cannot satisfy the reliability of the system. After data have been processed by the submodule, the fuzzy weighted fusion submodule fuses multiple inputs of data.

**Figure 11:**
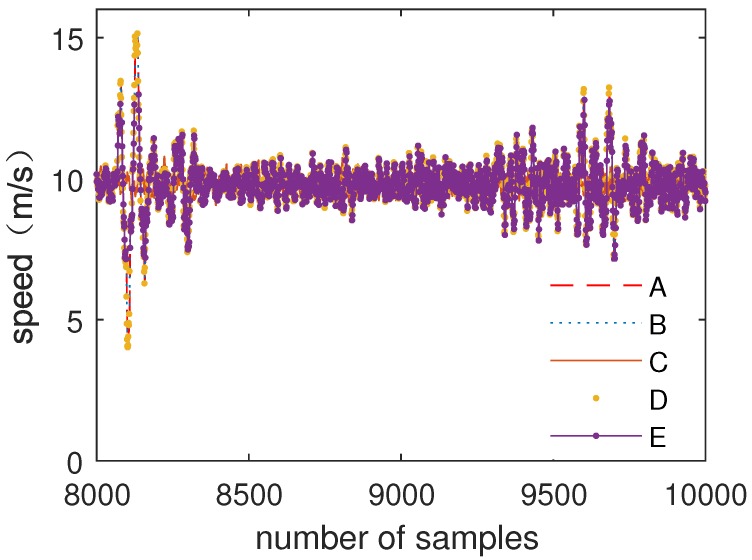
The comparison of speed data and data fusion.

Figure 11 shows the comparison of multiple inputs of speed data (A–D) and data processed by the fuzzy weighted fusion submodule (E).

**Figure 12:**
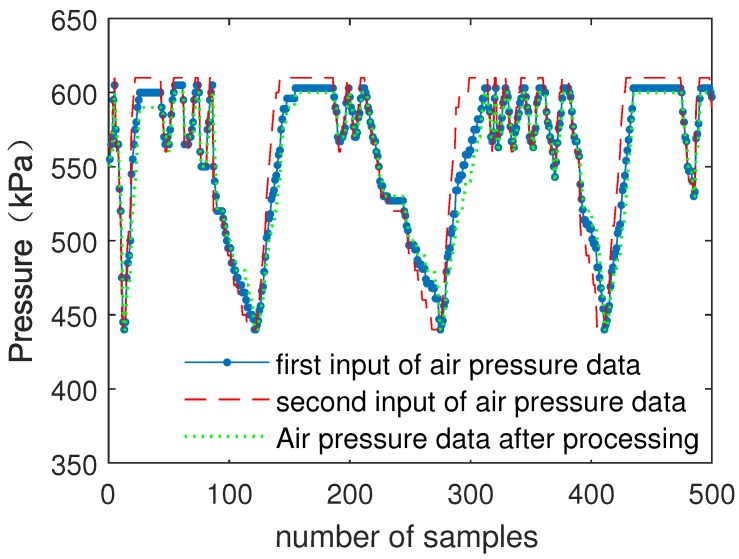
The comparison of air pressure data and data fusion.

Figure 12 shows the comparison of multiple inputs of air pressure data and data processed by the fuzzy weighted fusion submodule.

As can be seen from Figure 11 and Figure 12, after data filtering and data fusion, accurate data are obtained by multiple inputs of data. The results show that the fuzzy weighted fusion submodule improves the robustness and accuracy of data.

In order to verify the fault tolerance of the fuzzy processing model, one of inputs of speed data and air pressure data is in the state of error to verify whether the fuzzy processing model can output the correct data by other inputs.

**Figure 13:**
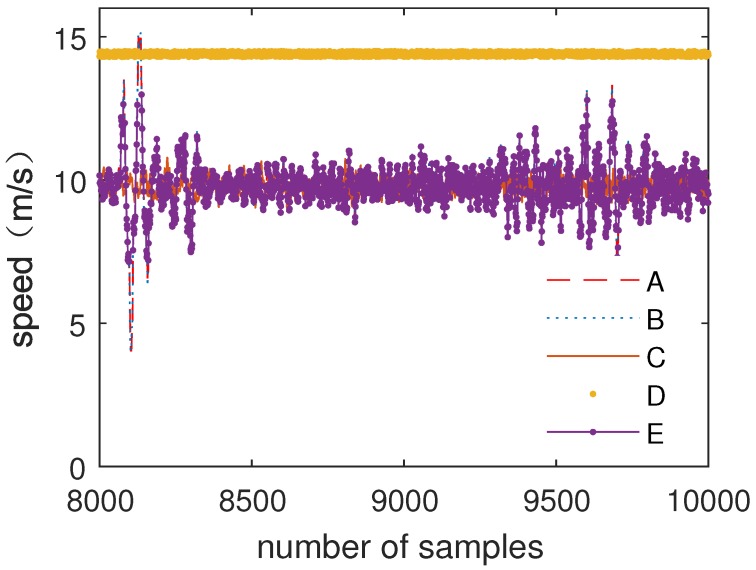
Speed data processing with one error input.

Figure 13 shows the comparison of multiple inputs of speed data (A–D) and data processed by the fuzzy processing model (E).

**Figure 14:**
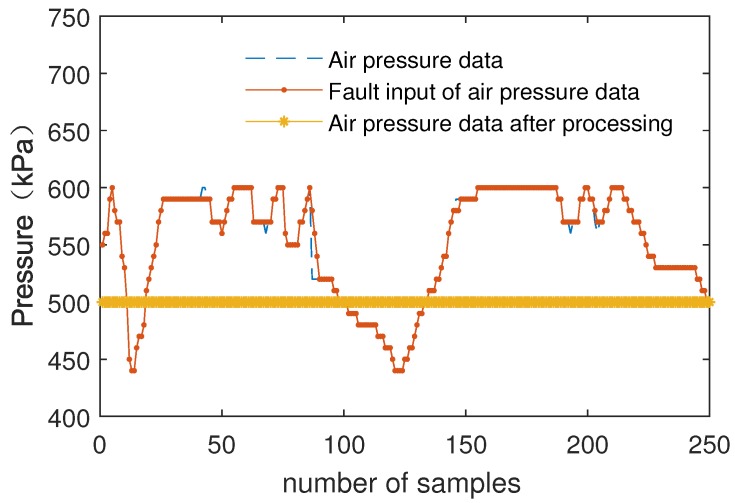
Air pressure data processing with one error input.

Figure 14 shows the comparison of multiple inputs of air pressure data and data processed by the fuzzy processing model.

As can be seen from Figure 13 and Figure 14, the error input barely affects the result, which is processed by the fuzzy processing model. The fuzzy processing model can process data correctly with one error input or even multiple error inputs. The results show that the fuzzy processing model has strong robustness and can further improve the accuracy of data.

According to the above analysis, it proves that the fuzzy processing model can effectively reduce the non-strict multi-sensor data problems, improve the data processing capability, as well as the system efficiency. In addition, the fuzzy processing model provides a high level of robustness.

In this section, the speed data and the air pressure data are modeled by the fuzzy processing model. Based on previous analysis and verification, the results show that the non-strict multi-sensor data problems of dynamic data are improved, indicating that the fuzzy processing model can improve the data processing capability and system efficiency. However, the non-strict problem of the safety computer in the train control system is not only limited to these two kinds of multi-sensor data, but also has corresponding problems for multi-sensor data such as bearing temperature and train safety operation condition evaluation. These data play important roles in the train control system. This needs further study. It is worth noting that the method studied in this paper can be applied to other heterogeneous redundant structure data.

There are several important things to mention. Firstly, The method studied in this paper can be applied to other heterogeneous redundant structure data. One kind of data may come from different sensors, and the data of different sensors have different data features. Therefore, it is necessary to collect a large amount of data and establish a database to analyze the data features before they can be used. Secondly, the membership functions of different data are totally different. The membership function plays a decisive role in the simulation, so we may need to adjust the parameter in the data analysis. Then, the sensor fusion data can be obtained by using the weighted fusion formula and the fuzzy attribute matrix.

A large amount of analog data and digital data will be generated in the operation of the train control system; both of them are essential. There are some important digital data problems in the train control system, but the method of fuzzy theory in this paper is only applicable to analog data. Therefore, it is important to make further efforts in that direction.

## 5. Conclusions

Traditional safety computers in the train control system only compare multiple input data and operate based on the sameness of data, and all types of data perform the same operation. The architecture is simple, but has non-strict multi-sensor data problems and a low efficiency in performance. In this paper, the non-strict multi-sensor data problems that exist in traditional safety computers are analyzed. The multi-sensor input data are classified into three types based on data features and safety computer features. The feature extraction of train control data is discussed in detail, and data features are selected from three aspects of data features to reflect the operating state of the train control system accurately. The non-strict multi-sensor data problems of the safety computer can only be caused by dynamic data; therefore, this paper proposes a fuzzy processing model that only processes dynamic data in order to improve the data processing capability and system efficiency. Then, an improved onboard safety computer platform that combines a double two-vote-two onboard safety computer with a fuzzy processing model for processing dynamic data is proposed. The fuzzy processing model is divided into two parts: improved fuzzy decision tree and fuzzy weighted fusion. Finally, the fuzzy processing model is verified based on the speed and air pressure data. Verification results indicate that the fuzzy processing model can effectively reduce the non-strict multi-sensor data problems and improve the system’s efficiency and robustness on the premise of ensuring the reliability of the input data.

## Abbreviations

The following abbreviation is used in this manuscript:

FID3	fuzzy iterative dichotomizer 3
